# Supplementary figures and images for: How long is a piece of loop?
Source: PeerJ. 2013 Feb 12;1:e1. doi: 10.7717/peerj.1 (PMC3628373; doi:10.7717/peerj.1)

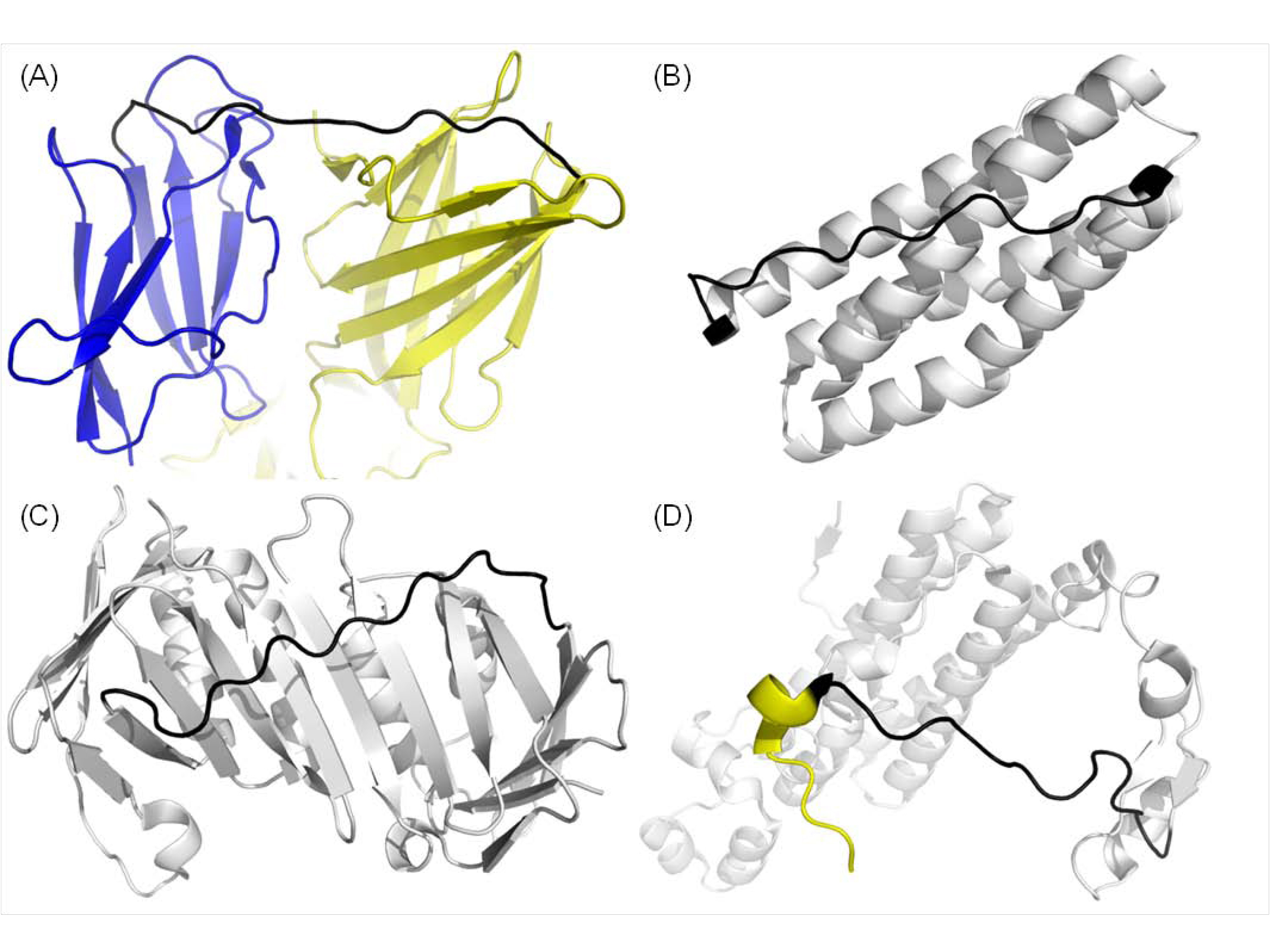

Supplement: Fig. S1 — There are mainly four classes of exceptionally long and stretched loops. (A) Domain linker (1OK8, Chain A 291-305), (B) local secondary structure packing (1YV1, Chain A 88-103), (C) global protein fold (3F1W, Chain A 118-134) and (D) short secondary structures at terminal regions (1O9I, Chain A 243-258). [file peerj-01-1-s001.jpg]

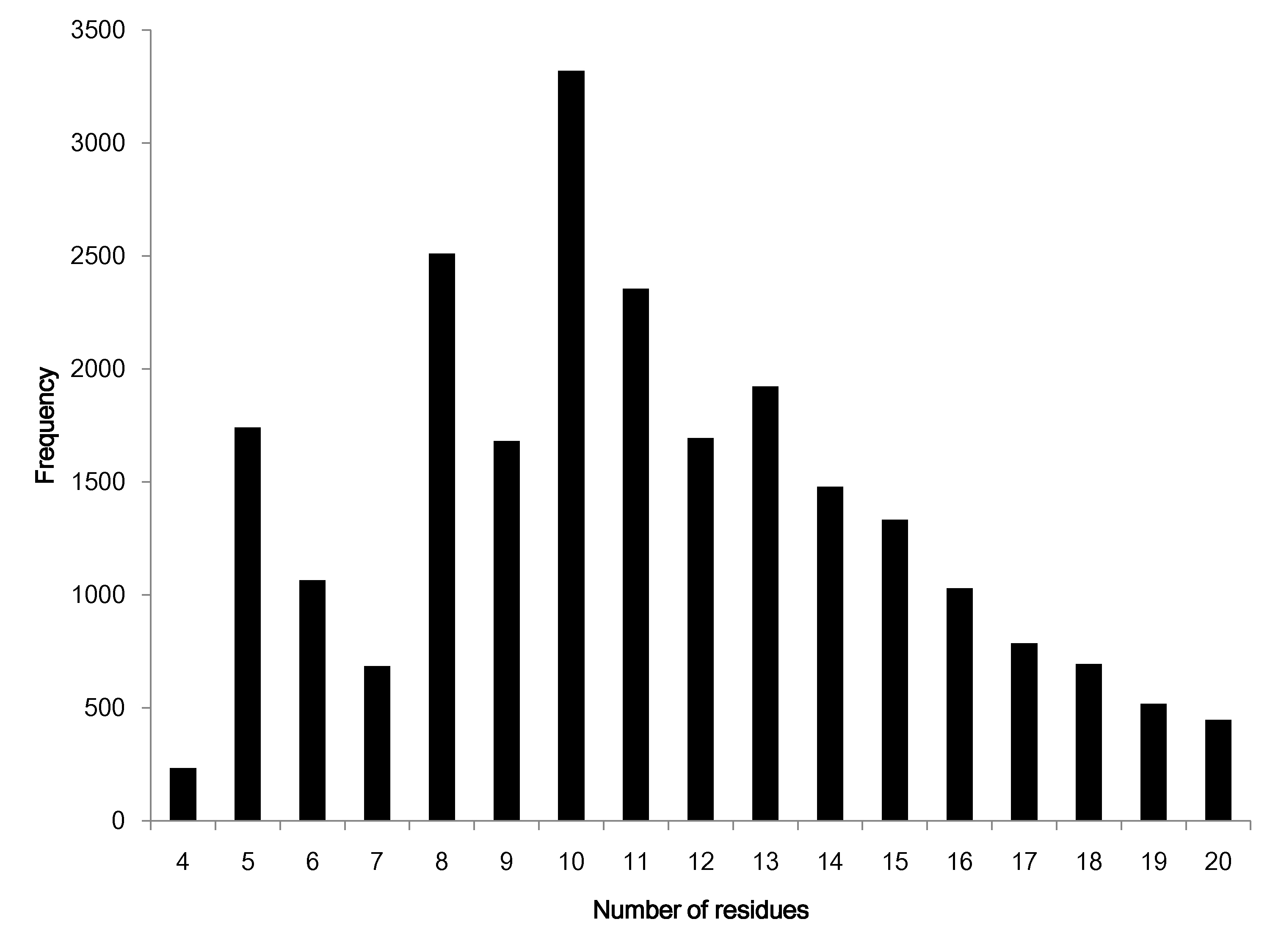

Supplement: Fig. S2 [file peerj-01-1-s002.jpg]
