# Supplementary material for: How long is a piece of loop?
Source: PeerJ. 2013 Feb 12;1:e1. doi: 10.7717/peerj.1 (PMC3628373; doi:10.7717/peerj.1)
Supplement: Table S1 — This test set only consists of loops of 8 residues in length. N and C columns are secondary structure elements (E: strand, H: helix). [file peerj-01-1-s003.docx]

| Code | Chain | Start | Length | Sequence | N | C | Span | Stretch |
| --- | --- | --- | --- | --- | --- | --- | --- | --- |
| 1R8S | A | 158 | 8 | TCATSGDG | E | H | 10.67 | 0.21 |
| 2QF4 | A | 243 | 8 | HSTDYLTR | E | E | 15.47 | 0.22 |
| 1RI6 | A | 207 | 8 | LKDPHGNI | E | E | 15.89 | 0.22 |
| 2HSJ | A | 188 | 8 | TTDGLHLS | H | H | 11.86 | 0.22 |
| 2FSR | A | 84 | 8 | HGPLFPEK | E | E | 9.18 | 0.23 |
| 2ZUX | A | 494 | 8 | WWDGDLLR | E | E | 11.10 | 0.23 |
| 1YOC | A | 53 | 8 | LNHIGTVH | H | H | 14.34 | 0.23 |
| 3CQL | A | 160 | 8 | TPQAPKPS | H | H | 13.00 | 0.23 |
| 1FN9 | A | 262 | 8 | TPILGKMP | E | E | 8.09 | 0.24 |
| 2I5V | O | 149 | 8 | GIKSDGSG | E | E | 9.68 | 0.24 |
| 2HC1 | A | 1764 | 8 | PGNNFRRE | E | E | 8.90 | 0.24 |
| 2GMN | A | 50 | 8 | VGTDGIAV | E | E | 9.24 | 0.25 |
| 2WHJ | A | 161 | 8 | AAGWGQYP | E | H | 14.93 | 0.25 |
| 2G2C | A | 124 | 8 | TGRDDHAA | E | E | 14.28 | 0.25 |
| 2EHZ | A | 194 | 8 | HCNARDHS | E | E | 8.01 | 0.25 |
| 3ENU | A | 102 | 8 | DNHNYRDE | E | E | 12.78 | 0.25 |
| 1VRM | A | 86 | 8 | FSFTDERS | H | H | 15.66 | 0.25 |
| 2Q0I | A | 156 | 8 | AHGHSDDH | E | E | 15.73 | 0.25 |
| 2QED | A | 107 | 8 | TPGHTLGH | E | E | 6.93 | 0.25 |
| 2VSM | A | 527 | 8 | DSNQTAEN | E | E | 8.32 | 0.25 |
| 1KNM | A | 63 | 8 | AGTSNGSK | E | E | 8.27 | 0.25 |
| 2E7Z | A | 9 | 8 | CQSCDINC | E | E | 10.58 | 0.26 |
| 1LZL | A | 203 | 8 | FVDTPLWH | H | H | 13.78 | 0.26 |
| 1UPS | A | 349 | 8 | IENQTGYI | E | E | 16.03 | 0.26 |
| 3H0N | A | 147 | 8 | CNAHRCDR | E | E | 10.02 | 0.26 |
| 3FJZ | A | 143 | 8 | LEQENYPP | E | E | 14.43 | 0.26 |
| 2HO3 | A | 248 | 8 | NTIEHIRS | E | E | 4.38 | 0.26 |
| 2QZU | A | 405 | 8 | KFDNHATG | E | E | 12.31 | 0.26 |
| 1Q35 | A | 220 | 8 | FPSGEHGT | E | E | 9.61 | 0.27 |
| 2FNO | A | 12 | 8 | YWPVPFRG | E | H | 11.63 | 0.27 |
| 1E9G | A | 60 | 8 | TKEETLNP | E | E | 12.36 | 0.27 |
| 2ZKM | X | 642 | 8 | FEFNGQSG | H | E | 10.03 | 0.27 |
| 1G0S | A | 132 | 8 | ASPGGTSE | E | E | 14.04 | 0.28 |
| 2CL2 | A | 121 | 8 | GVNDQSPN | E | E | 6.08 | 0.28 |
| 1OEW | A | 187 | 8 | VSTKQGFW | E | E | 10.72 | 0.28 |
| 1J0P | A | 71 | 8 | DKGTKFKS | H | H | 9.13 | 0.29 |
| 1VHE | A | 306 | 8 | HLTANGVP | H | E | 6.70 | 0.29 |
| 2OSX | A | 102 | 8 | EPAPGVYD | H | H | 8.46 | 0.29 |
| 1PJX | A | 253 | 8 | GPDGGQPK | E | E | 12.41 | 0.29 |
| 1Y43 | B | 14 | 8 | DGDTCETA | E | E | 10.16 | 0.30 |
| 1W78 | A | 147 | 8 | VGLGGRLD | E | H | 9.19 | 0.30 |
| 1QFM | A | 639 | 8 | DHDDRVVP | E | H | 7.35 | 0.30 |
| 1M2D | A | 89 | 8 | HLKGGEPV | H | H | 15.94 | 0.31 |
| 1PJX | A | 220 | 8 | IPGTHEGG | E | E | 11.88 | 0.31 |
| 3GRD | A | 37 | 8 | AEGFPYGG | E | E | 8.88 | 0.31 |
| 3BON | A | 156 | 8 | PSADIIQF | E | E | 12.51 | 0.31 |
| 1JI1 | A | 250 | 8 | STANGPKG | H | E | 14.52 | 0.31 |
| 1S9U | A | 23 | 8 | YSPESHET | H | H | 13.12 | 0.32 |
| 3BBB | A | 109 | 8 | CIQVGRNI | H | E | 14.72 | 0.32 |
| 1C5E | A | 49 | 8 | WDGTTDGA | E | E | 10.29 | 0.33 |
| 1OR0 | B | 120 | 8 | IRSSVHGP | E | E | 15.03 | 0.33 |
| 2W5N | A | 318 | 8 | QALGDASA | E | E | 13.07 | 0.33 |
| 1JND | A | 344 | 8 | SDPTKRFG | E | E | 12.59 | 0.34 |
| 2OLM | A | 57 | 8 | RGLNPPHR | H | E | 10.14 | 0.34 |
| 3DAQ | A | 245 | 8 | SVDINPIP | H | H | 7.87 | 0.34 |
| 1OK0 | A | 59 | 8 | GYIGSHGH | E | E | 9.18 | 0.35 |
| 2I5V | O | 204 | 8 | TDSSAATK | E | E | 15.44 | 0.35 |
| 2O4U | X | 1250 | 8 | LNPCWCPT | E | E | 15.26 | 0.35 |
| 1UWF | A | 46 | 8 | NDYPETIT | E | E | 12.77 | 0.35 |
| 2PVB | A | 18 | 8 | CSAADSFK | H | H | 7.61 | 0.36 |
| 2Z0J | A | 141 | 8 | AGKEGRAG | E | H | 12.55 | 0.36 |
| 1QW9 | A | 351 | 8 | QLVNVIAP | E | E | 11.96 | 0.36 |
| 2J2J | A | 502 | 8 | NKPKGVAT | E | E | 10.56 | 0.36 |
| 1JI1 | A | 468 | 8 | LSNHDITR | E | H | 9.58 | 0.36 |
| 2PRV | A | 61 | 8 | LGYNFDHA | E | H | 14.22 | 0.36 |
| 1RU4 | A | 300 | 8 | QNNNAGGV | E | E | 6.27 | 0.37 |
| 3BIO | A | 119 | 8 | IASGWDPG | E | H | 13.78 | 0.38 |
| 1WDP | A | 245 | 8 | TGFFKSNG | H | H | 13.72 | 0.38 |
| 2CKK | A | 80 | 8 | LNGGYRGN | E | E | 15.85 | 0.38 |
| 3E9K | A | 149 | 8 | FKPTPKRY | H | E | 12.89 | 0.38 |
| 1ZJA | A | 116 | 8 | RASKDNPY | H | H | 14.03 | 0.38 |
| 1GKM | A | 138 | 8 | GSVGASGD | E | H | 5.59 | 0.38 |
| 2H98 | A | 195 | 8 | YPVSQKPN | E | H | 6.32 | 0.38 |
| 2OBL | A | 322 | 8 | LESDNVND | E | H | 16.00 | 0.39 |
| 2CXN | A | 412 | 8 | TQHPIRKG | E | H | 13.07 | 0.39 |
| 2P02 | A | 166 | 8 | DETEECMP | E | H | 9.32 | 0.39 |
| 2ZKM | X | 395 | 8 | AFKTSPYP | H | E | 10.64 | 0.39 |
| 3C1Q | A | 123 | 8 | GDYPHVFD | H | H | 8.69 | 0.39 |
| 2CN3 | A | 349 | 8 | NAWWPDEY | E | E | 11.66 | 0.39 |
| 1QL0 | A | 177 | 8 | NNSPAVNH | E | E | 9.10 | 0.40 |
| 1ITX | A | 162 | 8 | GGWTWSNR | E | H | 7.24 | 0.41 |
| 1ODM | A | 175 | 8 | FKPDDTLA | H | E | 7.27 | 0.41 |
| 2VLQ | B | 92 | 8 | QVGGRKVY | H | E | 9.41 | 0.41 |
| 1VHE | A | 17 | 8 | AKGIPGNE | H | H | 6.09 | 0.41 |
| 2Z3H | A | 21 | 8 | IPISEDYS | H | E | 11.10 | 0.41 |
| 1WUI | S | 235 | 8 | KFNQTNWP | H | H | 9.32 | 0.41 |
| 2BJK | A | 317 | 8 | FQGQKCSA | H | E | 7.39 | 0.42 |
| 3SIL | A | 246 | 8 | RNSGLRRS | E | E | 7.62 | 0.42 |
| 2J2J | A | 367 | 8 | TGPGPSIN | E | E | 5.14 | 0.43 |
| 3B8D | A | 152 | 8 | KIGEHTPS | E | H | 7.37 | 0.43 |
| 2GKE | A | 164 | 8 | VDDIQTAN | E | H | 11.05 | 0.43 |
| 2C71 | A | 631 | 8 | DVQPEPHP | E | H | 7.84 | 0.44 |
| 1GUD | A | 66 | 8 | APLSSVNL | E | H | 9.03 | 0.44 |
| 1JND | A | 15 | 8 | REGLGKLL | H | H | 10.50 | 0.44 |
| 1GKM | A | 108 | 8 | SRGFSGIR | H | H | 9.73 | 0.45 |
| 1V7W | A | 355 | 8 | AISVPHAN | H | H | 10.87 | 0.45 |
| 1UCS | A | 26 | 8 | VVTPMGIP | E | H | 5.73 | 0.45 |
| 3BFV | A | 1158 | 8 | TPPVNTVT | E | H | 6.29 | 0.45 |
| 2CNQ | A | 233 | 8 | DEVLTPDS | E | E | 5.97 | 0.45 |
| 1CHD | A | 280 | 8 | LTGMGNDG | E | H | 10.82 | 0.45 |
| 1DS1 | A | 164 | 8 | ADHERTAA | E | E | 11.26 | 0.46 |
| 1RU4 | A | 214 | 8 | KQKQGPGN | E | E | 8.83 | 0.46 |
| 1Q6Z | A | 387 | 8 | LNMRNPGS | H | E | 10.56 | 0.46 |
| 1FJ2 | A | 199 | 8 | EGMMHSSC | E | H | 10.99 | 0.46 |
| 2AQ5 | A | 252 | 8 | TKHLEEPL | E | E | 8.68 | 0.47 |
| 2IW1 | A | 203 | 8 | GSDFGRKG | E | H | 7.98 | 0.47 |
| 1XRU | A | 239 | 8 | SPSWSIHS | E | E | 9.47 | 0.47 |
| 3DAQ | A | 106 | 8 | TPYYNKTN | E | H | 7.28 | 0.47 |
| 1UAS | A | 131 | 8 | NCNDAGRS | E | H | 7.96 | 0.48 |
| 1TJY | A | 91 | 8 | SAVSPDGL | E | H | 9.01 | 0.48 |
| 3FDY | A | 437 | 8 | TLFQPSHP | E | E | 9.73 | 0.48 |
| 2VHK | A | 104 | 8 | NIKGFNVP | E | E | 6.38 | 0.48 |
| 2OXG | A | 66 | 8 | DPAVSTNP | E | E | 10.12 | 0.49 |
| 1SU8 | A | 197 | 8 | TSMGCDAD | H | H | 8.77 | 0.49 |
| 2D81 | A | 52 | 8 | YSDVFNVG | H | E | 9.14 | 0.49 |
| 3CCD | A | 8 | 8 | ITAPNGLD | E | H | 7.42 | 0.49 |
| 1M55 | A | 154 | 8 | LLPKVQPE | H | E | 11.11 | 0.49 |
| 1ODM | A | 164 | 8 | LGKEENFF | H | H | 10.31 | 0.49 |
| 1GNL | A | 73 | 8 | TITNANFD | H | H | 4.76 | 0.49 |
| 1U4G | A | 105 | 8 | HYGRSVEN | E | E | 9.38 | 0.50 |
| 2VZP | A | 61 | 8 | NGTTTSRP | E | E | 6.53 | 0.50 |
| 1L7A | A | 294 | 8 | RYFGHEYI | E | H | 11.08 | 0.50 |
| 2IW1 | A | 277 | 8 | PAYQEAAG | E | H | 7.74 | 0.50 |
| 1KWG | A | 284 | 8 | YARTGHPD | H | H | 10.94 | 0.50 |
| 1NKG | A | 246 | 8 | LDIKGYVA | H | H | 7.75 | 0.51 |
| 2EIY | B | 211 | 8 | EHSVNLEG | E | H | 10.08 | 0.52 |
| 2CWS | A | 286 | 8 | QDNTSTGG | E | E | 6.69 | 0.52 |
| 1P3D | A | 84 | 8 | SSAIKDDN | E | H | 11.50 | 0.52 |
| 2FI1 | A | 12 | 8 | DLGGTLLD | E | H | 9.78 | 0.52 |
| 2I0K | A | 255 | 8 | LTNLGRCF | H | E | 8.86 | 0.52 |
| 2IW1 | A | 87 | 8 | FNKMPGLD | E | E | 8.42 | 0.52 |
| 3FVS | A | 62 | 8 | QYTKTFGY | H | H | 11.19 | 0.53 |
| 1MDL | A | 91 | 8 | FCLAGYTG | H | H | 9.75 | 0.53 |
| 2VK2 | A | 65 | 8 | APVVATGW | E | H | 8.22 | 0.53 |
| 3BMV | A | 472 | 8 | ASDGSVTP | E | E | 6.93 | 0.53 |
| 2R9F | A | 107 | 8 | ICQGALGD | H | H | 10.20 | 0.54 |
| 2I7D | A | 10 | 8 | DMDGVLAD | E | H | 5.99 | 0.54 |
| 3E9K | A | 436 | 8 | APVPLYNS | E | H | 8.87 | 0.54 |
| 3CUZ | A | 236 | 8 | FNLPRGTI | H | E | 7.37 | 0.54 |
| 3D32 | A | 71 | 8 | HLRAEDAL | H | E | 10.00 | 0.54 |
| 2QV8 | A | 159 | 8 | KGQEPDEQ | E | E | 10.44 | 0.55 |
| 2OXN | A | 7 | 8 | DVAGYELS | E | H | 8.50 | 0.55 |
| 1UFY | A | 50 | 8 | TEDLTSAF | E | H | 9.83 | 0.55 |
| 3H6J | A | 353 | 8 | HGLQRSPR | E | E | 9.11 | 0.55 |
| 2AQ5 | A | 171 | 8 | GPDVHPDT | E | E | 5.50 | 0.56 |
| 1CSH | A | 235 | 8 | HSDHEGGN | H | H | 8.95 | 0.56 |
| 1HT6 | A | 346 | 8 | NGITATSA | H | E | 7.18 | 0.57 |
| 1H97 | A | 97 | 8 | DHTSRKVT | H | H | 11.50 | 0.57 |
| 2VFR | A | 402 | 8 | DPAGKFTN | H | H | 6.31 | 0.57 |
| 2BWR | A | 331 | 8 | GWRVDKHP | H | E | 8.80 | 0.58 |
| 1VLA | A | 30 | 8 | GGKDAAPR | H | H | 7.27 | 0.58 |
| 2V8T | A | 236 | 8 | FSPEDYRD | E | H | 10.68 | 0.58 |
| 1B8O | A | 195 | 8 | LGGPNFET | E | H | 9.07 | 0.58 |
| 1Y4W | A | 504 | 8 | IFPSSDAV | E | E | 11.08 | 0.58 |
| 1WKR | A | 147 | 8 | FEPTTSES | E | E | 11.06 | 0.59 |
| 2UYT | A | 128 | 8 | GIQFLPFN | H | H | 11.33 | 0.59 |
| 1T3Y | A | 102 | 8 | KEVVQNFA | H | E | 10.84 | 0.59 |
| 1GWM | A | 104 | 8 | VDFDLPFD | E | E | 8.53 | 0.59 |
| 2E6F | A | 86 | 8 | LHDYSKKP | H | E | 9.94 | 0.59 |
| 3G7R | A | 103 | 8 | RSPGFRGC | H | H | 10.12 | 0.59 |
| 1YAC | A | 137 | 8 | TDASGTFN | E | H | 7.05 | 0.60 |
| 1GA6 | A | 304 | 8 | NSNSLGFP | H | H | 8.92 | 0.60 |
| 3E2D | A | 220 | 8 | FAYSGMDD | E | H | 4.84 | 0.61 |
| 1HDH | A | 13 | 8 | DDLGFSDI | E | H | 9.64 | 0.61 |
| 3F1L | A | 156 | 8 | RQGRANWG | H | H | 11.14 | 0.61 |
| 1RQB | A | 215 | 8 | HCHSTTGV | E | H | 10.08 | 0.62 |
| 1Q0R | A | 272 | 8 | PGMGHALP | E | H | 9.12 | 0.62 |
| 3G28 | A | 156 | 8 | IMQGPSES | H | H | 9.30 | 0.62 |
| 1PX0 | A | 206 | 8 | TALQRLGT | H | H | 11.75 | 0.62 |
| 1NKG | A | 98 | 8 | TAEPSIGE | E | E | 10.15 | 0.62 |
| 2HZL | A | 236 | 8 | PGWWEGGP | E | E | 7.93 | 0.62 |
| 1EEX | A | 133 | 8 | RARRTPSQ | H | E | 10.95 | 0.62 |
| 2GGC | A | 138 | 8 | RMVKPGIN | H | H | 9.52 | 0.62 |
| 2D81 | A | 80 | 8 | MYNGYPSI | H | H | 10.23 | 0.62 |
| 1GA6 | A | 17 | 8 | YDASSAPT | H | E | 7.03 | 0.63 |
| 2GAI | A | 457 | 8 | LGIGRPST | H | H | 9.91 | 0.64 |
| 1DK8 | A | 212 | 8 | QLIDPAMF | H | H | 10.54 | 0.64 |
| 1GQI | A | 696 | 8 | TYVPEPWH | H | H | 8.82 | 0.64 |
| 2WHJ | A | 88 | 8 | HDATGRDS | E | H | 11.60 | 0.64 |
| 2JE8 | A | 395 | 8 | WGGGTYEN | E | H | 8.62 | 0.64 |
| 1VLP | A | 177 | 8 | FGTRRRRS | E | H | 9.15 | 0.65 |
| 1UAS | A | 247 | 8 | GCDVRSMS | E | H | 10.24 | 0.65 |
| 1H16 | A | 327 | 8 | FSGDPIWA | H | E | 10.56 | 0.66 |
| 3FDY | A | 476 | 8 | RTEPKEEN | E | E | 10.09 | 0.66 |
| 3H7C | X | 120 | 8 | WNFNAWGG | E | H | 10.67 | 0.66 |
| 2V03 | A | 55 | 8 | RGEIKPGD | H | E | 9.60 | 0.66 |
| 1ATZ | A | 1038 | 8 | TDVSVDSV | E | H | 10.95 | 0.67 |
| 2OIZ | A | 416 | 8 | EGAAEASL | E | E | 11.71 | 0.68 |
| 1O7Q | A | 308 | 8 | NDIEAQWH | H | H | 11.21 | 0.68 |
| 3D03 | A | 49 | 8 | GDIVNCGR | E | H | 3.96 | 0.68 |
| 2VBK | A | 243 | 8 | SYDSDTIG | E | E | 9.01 | 0.68 |
| 2RFQ | A | 177 | 8 | DVWNVVGL | E | H | 11.02 | 0.68 |
| 2ZUX | A | 358 | 8 | AGQGNHNL | H | E | 10.08 | 0.68 |
| 3DQP | A | 70 | 8 | VSGSGGKS | E | H | 11.74 | 0.68 |
| 2PQC | A | 158 | 8 | GPKTPTPI | E | E | 9.98 | 0.68 |
| 1LFW | A | 265 | 8 | GQGAHASA | E | H | 10.47 | 0.68 |
| 2E6F | A | 18 | 8 | AAGVLCST | E | H | 6.07 | 0.68 |
| 1PQ7 | A | 170 | 8 | GTSAITNQ | H | E | 7.53 | 0.69 |
| 3BVX | A | 203 | 8 | IAPFGHSP | E | H | 9.08 | 0.69 |
| 3DG9 | A | 188 | 8 | CGGLLTLD | E | H | 8.11 | 0.70 |
| 1Y93 | A | 119 | 8 | NNYTPDMN | E | H | 6.47 | 0.70 |
| 2WAA | A | 271 | 8 | TEGAILNG | E | H | 11.66 | 0.70 |
| 1E58 | A | 205 | 8 | LNIPTGVP | H | E | 11.13 | 0.70 |
| 16PK | A | 339 | 8 | GPMGVFEM | E | H | 10.61 | 0.70 |
| 1IOM | A | 83 | 8 | KRYPVSAH | H | H | 10.19 | 0.70 |
| 2BHU | A | 524 | 8 | DPVLHNRQ | H | H | 8.52 | 0.70 |
| 2VFR | A | 62 | 8 | SLAGLPSV | E | E | 9.24 | 0.71 |
| 3GMV | X | 86 | 8 | NRTALGMT | H | H | 9.25 | 0.71 |
| 1SU8 | A | 330 | 8 | DYQCIQPS | E | H | 10.65 | 0.71 |
| 1Q0R | A | 126 | 8 | LGGGLDID | E | H | 10.76 | 0.71 |
| 2ZUX | A | 395 | 8 | TGLGHGDA | E | E | 10.97 | 0.72 |
| 1DYP | A | 131 | 8 | KGASTFPG | E | E | 8.88 | 0.72 |
| 1H4A | X | 158 | 8 | GATNARVG | H | E | 6.84 | 0.72 |
| 1TBF | A | 741 | 8 | KNQFNLED | H | H | 10.61 | 0.72 |
| 1QFM | A | 578 | 8 | VGVMDMLK | E | H | 8.17 | 0.72 |
| 1U4G | A | 92 | 8 | GTSPLTHK | H | E | 11.47 | 0.73 |
| 1F5V | A | 90 | 8 | CPDAQLGL | H | H | 10.89 | 0.73 |
| 1H4A | X | 69 | 8 | MGLSDSVR | H | E | 11.27 | 0.73 |
| 3B8D | A | 258 | 8 | RTVPPAVT | H | E | 2.43 | 0.74 |
| 1PN2 | A | 231 | 8 | TGIVFPGE | E | E | 4.23 | 0.74 |
| 3H09 | A | 443 | 8 | KVHNPKSD | E | E | 5.80 | 0.75 |
| 3EIX | A | 222 | 8 | LGFKNALS | H | H | 10.10 | 0.75 |
| 2ZQ0 | A | 507 | 8 | HEATRPTG | E | H | 10.12 | 0.75 |
| 2V3Z | A | 419 | 8 | LTASVVKK | E | H | 11.22 | 0.76 |
| 1GCI | A | 19 | 8 | RGLTGSGV | H | E | 10.57 | 0.76 |
| 2BHU | A | 381 | 8 | GHPSDALE | E | H | 6.82 | 0.77 |
| 2H1V | A | 286 | 8 | RPEMPNAK | E | H | 7.63 | 0.77 |
| 3BMV | A | 365 | 8 | YMTGNGDP | H | H | 7.80 | 0.77 |
| 1GNL | A | 396 | 8 | MAGCDGRA | E | H | 5.76 | 0.77 |
| 1OXX | K | 329 | 8 | DHPIHSGE | E | E | 11.36 | 0.77 |
| 1PJX | A | 297 | 8 | WQRNGKKQ | E | H | 10.21 | 0.77 |
| 2CYJ | A | 55 | 8 | KYLVEDFD | H | E | 8.45 | 0.77 |
| 7A3H | A | 187 | 8 | DNQLADPN | H | E | 10.74 | 0.78 |
| 1ODZ | A | 142 | 8 | SHFDNPKT | E | H | 11.58 | 0.78 |
| 1NKG | A | 470 | 8 | PSGTIVAG | E | E | 10.78 | 0.78 |
| 2JG0 | A | 203 | 8 | LSRSQPPF | H | H | 5.29 | 0.78 |
| 2GZQ | A | 61 | 8 | FSEEPPED | E | E | 6.55 | 0.79 |
| 1K2X | A | 133 | 8 | ARGMERVS | H | H | 10.92 | 0.79 |
| 1OK0 | A | 38 | 8 | EDDTEGLC | E | E | 8.89 | 0.80 |
| 1K0M | A | 196 | 8 | GFTIPEAF | H | H | 9.70 | 0.80 |
| 3HID | A | 101 | 8 | SEACPLIL | E | H | 14.19 | 0.80 |
| 2HO3 | A | 228 | 8 | KNITSNLP | E | E | 4.57 | 0.80 |
| 3GN6 | A | 108 | 8 | AGTQPYFY | H | E | 15.51 | 0.80 |
| 3BPT | A | 172 | 8 | IGLFPDVG | H | H | 9.35 | 0.81 |
| 1LLF | A | 325 | 8 | DGKYASVP | H | E | 15.42 | 0.81 |
| 1VLP | A | 39 | 8 | RSSQLTFN | E | H | 9.05 | 0.81 |
| 3GVE | A | 59 | 8 | NGDLIQGN | E | H | 9.81 | 0.81 |
| 2EIY | B | 67 | 8 | LRMEIPFA | H | H | 14.45 | 0.81 |
| 1GNL | A | 509 | 8 | PTLPAFLS | E | H | 8.30 | 0.82 |
| 1OQ1 | A | 52 | 8 | CPETFPDG | E | E | 11.17 | 0.82 |
| 1R8S | E | 132 | 8 | LHEFTDLN | H | H | 13.62 | 0.82 |
| 2AYH | A | 194 | 8 | GSYNGANP | H | E | 15.21 | 0.82 |
| 3DEL | B | 130 | 8 | GENKHPLP | E | H | 4.43 | 0.82 |
| 1MUN | A | 131 | 8 | GKHFPILN | H | H | 12.66 | 0.84 |
| 1UAS | A | 182 | 8 | TTGDIADN | E | H | 7.85 | 0.84 |
| 2E6F | A | 68 | 8 | SMGLPNLG | E | H | 6.11 | 0.84 |
| 1HP1 | A | 82 | 8 | GGDINTGV | E | H | 8.71 | 0.84 |
| 1J1N | A | 149 | 8 | LNLKPPQN | H | H | 16.45 | 0.84 |
| 2V3V | A | 153 | 8 | GKDEPMGT | H | H | 16.27 | 0.85 |
| 1UWF | A | 85 | 8 | PTTSETPR | E | E | 15.13 | 0.85 |
| 2QMQ | A | 170 | 8 | NIDPNAKG | E | H | 15.55 | 0.86 |
| 2OIZ | D | 131 | 8 | GKTACGRC | E | E | 10.18 | 0.86 |
| 1WUI | L | 379 | 8 | MGEPMETG | E | H | 9.17 | 0.86 |
| 1NZJ | A | 254 | 8 | LGQQAEAH | H | H | 16.33 | 0.86 |
| 2H98 | A | 146 | 8 | RLKITDPA | E | E | 9.34 | 0.86 |
| 1KA1 | A | 72 | 8 | EESSSGLS | E | H | 12.43 | 0.87 |
| 2ABK | A | 131 | 8 | GWPTIAVD | H | H | 15.67 | 0.87 |
| 1B5E | A | 141 | 8 | DGMSDFMC | H | E | 14.32 | 0.87 |
| 1DPE | A | 8 | 8 | EGSPEGFN | E | H | 14.78 | 0.87 |
| 2IYA | A | 235 | 8 | VGPTYGDR | E | H | 12.77 | 0.87 |
| 1NKG | A | 456 | 8 | GAYRGLGE | H | E | 6.01 | 0.88 |
| 1HP1 | A | 409 | 8 | IRDSIEAG | H | E | 14.15 | 0.88 |
| 2Z4U | A | 114 | 8 | KAINYNSQ | E | H | 11.88 | 0.89 |
| 3E03 | A | 148 | 8 | LAPPPSLN | E | H | 12.16 | 0.89 |
| 3BOE | A | 366 | 8 | HGSHTEKV | E | E | 13.72 | 0.89 |
| 1O9R | A | 76 | 8 | LGGTALGS | H | H | 16.08 | 0.89 |
| 2R8O | A | 633 | 8 | TTFGESAP | E | H | 7.48 | 0.89 |
| 3BWH | A | 233 | 8 | QNIQLLLN | H | H | 10.29 | 0.89 |
| 2PQC | A | 230 | 8 | GRGKLTGQ | E | E | 10.13 | 0.89 |
| 3BOE | A | 340 | 8 | GYPRPQFD | H | H | 16.72 | 0.90 |
| 1RQB | A | 403 | 8 | GKKPITQR | H | H | 38.52 | 0.90 |
| 3B8I | A | 254 | 8 | GAVASDLT | H | H | 35.67 | 0.90 |
| 1E4C | P | 105 | 8 | NRSIPAIH | H | H | 13.93 | 0.90 |
| 2VLQ | B | 81 | 8 | KGYSPATP | H | H | 8.22 | 0.91 |
| 3BZW | A | 122 | 8 | SSVPIGEW | H | E | 6.52 | 0.91 |
| 3B5M | A | 128 | 8 | VDPFFGFN | E | H | 10.64 | 0.91 |
| 1U5P | A | 1793 | 8 | SSEDYGRD | H | H | 32.01 | 0.91 |
| 1LVM | A | 4 | 8 | LFKGPRDY | H | H | 20.88 | 0.91 |
| 2P4H | X | 301 | 8 | AGFDFKYT | H | H | 14.11 | 0.91 |
| 2INU | A | 124 | 8 | GFGHGFFS | E | H | 12.64 | 0.91 |
| 2VUW | A | 644 | 8 | RFEHRDLH | H | H | 18.41 | 0.92 |
| 2LIS | A | 108 | 8 | KNMIPKYL | H | H | 17.44 | 0.92 |
| 1VKM | A | 234 | 8 | KIELEVEG | H | H | 36.00 | 0.92 |
| 3CXU | A | 249 | 8 | TGAQVKVP | H | E | 13.62 | 0.92 |
| 1CQX | A | 42 | 8 | VFNMAHQE | H | H | 34.09 | 0.92 |
| 2EZ9 | A | 336 | 8 | QVSEREST | H | H | 17.90 | 0.92 |
| 2HY7 | A | 202 | 8 | VGHGVDHN | E | H | 14.68 | 0.92 |
| 1NNF | A | 68 | 8 | AGLLAPIS | H | H | 9.24 | 0.92 |
| 3H87 | C | 41 | 8 | QTARVTVT | H | H | 16.46 | 0.93 |
| 3FHL | A | 299 | 8 | PGIAGNYG | E | H | 30.11 | 0.93 |
| 2IYA | A | 138 | 8 | PTFVAYEG | E | H | 12.17 | 0.93 |
| 3CUZ | A | 407 | 8 | APCDGERT | H | H | 11.08 | 0.93 |
| 2GMN | A | 146 | 8 | AFPAVKVD | H | E | 10.92 | 0.94 |
| 1H16 | A | 313 | 8 | RMVRFLRT | H | H | 9.72 | 0.94 |
| 2C2U | A | 119 | 8 | LGGSPLAA | H | H | 7.40 | 0.94 |
| 2WAA | A | 125 | 8 | LPAPVLPQ | E | E | 11.50 | 0.94 |
| 1VQZ | A | 240 | 8 | YGKSPEFN | H | E | 36.63 | 0.96 |
| 1QLW | A | 130 | 8 | LPDLFAAG | H | H | 15.82 | 0.96 |
| 1YKI | A | 202 | 8 | TLPKSRLP | H | H | 10.49 | 0.97 |
| 1L6P | A | 56 | 8 | ADVQLPQG | E | E | 30.43 | 0.97 |
| 1W2W | B | 327 | 8 | IIVEERNP | H | H | 16.32 | 0.98 |
| 2QMC | B | 537 | 8 | KPVMGDVN | E | E | 21.28 | 0.98 |
| 2Z26 | A | 248 | 8 | GTDSAPHA | E | H | 12.10 | 0.98 |
| 3CJE | A | 148 | 8 | GQANTIRH | H | E | 20.90 | 0.99 |
| 1VYR | A | 339 | 8 | KAELNPQR | H | H | 15.81 | 0.99 |
| 2IVF | A | 233 | 8 | GKMHMGYS | H | H | 23.60 | 0.99 |
| 1GWE | A | 224 | 8 | QQGVHNLS | E | H | 6.25 | 1.00 |
| 2JE8 | A | 556 | 8 | FGFQSFPE | E | H | 10.98 | 1.00 |
| 2PKF | A | 33 | 8 | VSLSFLVD | H | E | 15.48 | 1.02 |
| 1GK9 | A | 123 | 8 | GFTPKRWE | H | H | 13.80 | 1.05 |
