# Supplementary material for: How long is a piece of loop?
Source: PeerJ. 2013 Feb 12;1:e1. doi: 10.7717/peerj.1 (PMC3628373; doi:10.7717/peerj.1)
Supplement: Table S2 — This test set only consists of highly contracted loops (λ < 0.4). N and C columns are secondary structure elements (E: strand, H: helix). [file peerj-01-1-s004.docx]

| Code | Chain | Start | Length | Sequence | N | C | Span | Stretch |
| --- | --- | --- | --- | --- | --- | --- | --- | --- |
| 1B7M | A | 100 | 6 | VRDPQG | H | H | 4.24 | 0.27 |
| 1JMX | A | 101 | 6 | GRCHSG | H | H | 4.33 | 0.28 |
| 2ZEZ | A | 31 | 6 | AYGGTK | H | E | 4.45 | 0.29 |
| 1FGJ | A | 465 | 6 | WTYTEG | H | H | 4.50 | 0.29 |
| 1ORD | A | 231 | 6 | LAMAGG | H | E | 4.50 | 0.29 |
| 1U8E | A | 123 | 6 | QWRHSY | E | E | 4.52 | 0.29 |
| 1QLH | A | 282 | 6 | CQEAYG | H | E | 4.60 | 0.30 |
| 3GVD | A | 54 | 6 | ANPIMP | H | H | 4.62 | 0.30 |
| 2W20 | A | 592 | 6 | TNNVSH | E | H | 4.87 | 0.31 |
| 3GHA | A | 87 | 6 | FIDKGD | H | E | 5.04 | 0.32 |
| 1PZN | A | 248 | 6 | YIGRGA | H | H | 5.09 | 0.33 |
| 1J0A | A | 145 | 6 | KREGRK | H | E | 5.22 | 0.34 |
| 2RDX | A | 266 | 6 | ISNLGG | E | H | 5.24 | 0.34 |
| 1XPM | A | 108 | 6 | KEACYA | E | H | 5.30 | 0.34 |
| 1PPJ | D | 52 | 6 | VGVCYT | H | H | 5.34 | 0.34 |
| 1UA4 | A | 237 | 6 | LTKENY | H | H | 5.35 | 0.34 |
| 4CEL | A | 369 | 6 | DYYANM | E | H | 5.36 | 0.34 |
| 3C70 | A | 10 | 6 | HTICHG | E | H | 5.58 | 0.36 |
| 2A0U | A | 218 | 6 | ETRPWN | E | H | 5.59 | 0.36 |
| 1O04 | A | 193 | 6 | VAEQTP | E | H | 5.61 | 0.36 |
| 2JLG | A | 628 | 6 | LQYKWT | H | H | 5.64 | 0.36 |
| 1NH1 | A | 127 | 6 | LVKPYK | E | H | 5.72 | 0.37 |
| 1A8Q | A | 26 | 6 | HGWPLN | E | H | 5.79 | 0.37 |
| 1DMU | A | 286 | 6 | DKIGPR | H | E | 5.80 | 0.37 |
| 2C8J | A | 86 | 6 | KHIEPF | E | H | 5.85 | 0.38 |
| 3D3U | A | 346 | 6 | KRSKGG | H | E | 6.01 | 0.39 |
| 1X6L | A | 486 | 6 | FMSGEW | H | E | 6.04 | 0.39 |
| 2H57 | A | 71 | 6 | SGQGRY | E | H | 6.15 | 0.40 |
| 5CPA | A | 67 | 6 | GIHSRE | E | H | 6.18 | 0.40 |
| 1UUR | A | 644 | 7 | TAAAKKT | H | H | 5.21 | 0.29 |
| 1FRF | S | 231 | 7 | LFNQVNW | H | H | 5.53 | 0.30 |
| 1KCX | A | 370 | 7 | VATGKMD | H | H | 5.55 | 0.31 |
| 2BA1 | A | 131 | 7 | TKEEEMG | E | E | 5.58 | 0.31 |
| 1YIO | A | 56 | 7 | MRMPGMS | E | H | 5.59 | 0.31 |
| 1JGI | A | 340 | 7 | IGQDECQ | H | E | 5.69 | 0.31 |
| 3HJC | A | 309 | 7 | TSEFGWS | E | H | 5.71 | 0.31 |
| 1JHL | A | 36 | 7 | SNFNTGA | H | E | 5.73 | 0.32 |
| 2FPR | A | 149 | 7 | YDRETLN | E | H | 5.76 | 0.32 |
| 3C8U | A | 28 | 7 | GAPGSGK | E | H | 6.11 | 0.34 |
| 1VKB | A | 82 | 7 | EDCPSMY | H | E | 6.13 | 0.34 |
| 1J71 | A | 189 | 7 | VTSSVEL | E | E | 6.27 | 0.35 |
| 2AM1 | A | 411 | 7 | KTEDQDQ | E | H | 6.29 | 0.35 |
| 3ESM | A | 51 | 7 | TESETAA | E | E | 6.30 | 0.35 |
| 1RKI | A | 26 | 7 | YYYDNSV | H | E | 6.35 | 0.35 |
| 1A0I | A | 54 | 7 | SRVSKTI | E | H | 6.37 | 0.35 |
| 1RCE | A | 38 | 7 | NRDDVAL | H | H | 6.42 | 0.35 |
| 1IVY | A | 370 | 7 | DVDMACN | E | H | 6.42 | 0.35 |
| 4CMS | A | 184 | 7 | VTVQQYW | E | E | 6.44 | 0.36 |
| 1YOO | A | 256 | 7 | YSKNFGL | E | H | 6.53 | 0.36 |
| 1T0I | A | 94 | 7 | QYNWGYP | E | H | 6.74 | 0.37 |
| 3FEQ | A | 389 | 7 | VADEGAR | H | E | 6.75 | 0.37 |
| 3CC6 | A | 482 | 7 | DHPHIVK | H | E | 6.77 | 0.37 |
| 1KY3 | A | 158 | 7 | SAKNAIN | E | H | 6.79 | 0.37 |
| 1V6T | A | 35 | 7 | CGWHAGD | E | H | 6.82 | 0.38 |
| 2ZKI | A | 80 | 7 | TRYGNMA | E | H | 6.83 | 0.38 |
| 1IAY | A | 159 | 7 | LRWRTGV | H | E | 6.85 | 0.38 |
| 2YX9 | A | 442 | 7 | AIDGFTN | E | E | 6.86 | 0.38 |
| 2Z9S | A | 21 | 7 | MPDGQFK | E | E | 6.93 | 0.38 |
| 2A39 | A | 348 | 7 | WDQGGNM | E | H | 6.96 | 0.38 |
| 1KBB | A | 480 | 7 | SNSAEDP | E | E | 6.98 | 0.38 |
| 3FJU | B | 50 | 7 | QVWAHEK | H | E | 7.00 | 0.39 |
| 1TCA | A | 243 | 7 | STTGQAR | H | H | 7.04 | 0.39 |
| 1G59 | A | 180 | 7 | KSDGYPT | E | H | 7.06 | 0.39 |
| 2FP3 | A | 297 | 7 | HFQTAKC | H | H | 7.16 | 0.39 |
| 1U6Z | A | 106 | 7 | EKVIPYP | H | E | 7.18 | 0.40 |
| 1P2Z | A | 770 | 8 | NVAQCNMT | H | H | 4.05 | 0.19 |
| 1F6B | A | 178 | 8 | CSVLKRQG | E | H | 4.20 | 0.19 |
| 1QWO | A | 282 | 8 | YGAGNPLG | H | H | 4.23 | 0.20 |
| 2A5D | A | 154 | 8 | SCATSGDG | E | H | 4.37 | 0.20 |
| 2V5X | A | 29 | 8 | DSLAKIPK | H | H | 4.43 | 0.21 |
| 1ORD | A | 166 | 8 | GDLLIHEG | H | H | 4.45 | 0.21 |
| 1JXH | A | 12 | 8 | TDPSGGAG | E | H | 4.51 | 0.21 |
| 1SVI | A | 174 | 8 | FSSETKKG | E | H | 4.51 | 0.21 |
| 2H5E | A | 257 | 8 | GTALGNFG | E | H | 4.66 | 0.22 |
| 1M1Z | A | 416 | 8 | KRRHGRDK | H | H | 4.70 | 0.22 |
| 2HSJ | A | 188 | 8 | TTDGLHLS | H | H | 4.71 | 0.22 |
| 1PNF | A | 18 | 8 | AFGDGLSQ | E | E | 4.74 | 0.22 |
| 1Q4U | A | 57 | 8 | RQRWGLVH | H | H | 4.79 | 0.22 |
| 2EIS | A | 15 | 8 | TNHYGTLF | H | H | 4.84 | 0.22 |
| 2FN4 | A | 171 | 8 | ASAKLRLN | E | H | 4.85 | 0.22 |
| 3EUC | A | 185 | 8 | AQGSVCRS | H | E | 4.86 | 0.22 |
| 2B3Z | A | 42 | 8 | HLKYGEAH | E | H | 4.91 | 0.23 |
| 2FX5 | A | 236 | 8 | ATFYGAQC | H | H | 4.94 | 0.23 |
| 1CNS | A | 160 | 8 | TAQPPKPS | H | H | 4.97 | 0.23 |
| 3BWS | A | 298 | 8 | ASNQESGG | E | E | 4.99 | 0.23 |
| 5EAS | A | 130 | 8 | QDENGKFK | H | H | 5.00 | 0.23 |
| 1RTZ | A | 81 | 8 | GRGGPRTL | H | E | 5.03 | 0.23 |
| 2WBL | C | 158 | 8 | CSSKTQQN | E | H | 5.05 | 0.23 |
| 3E1E | A | 47 | 8 | TQQHGFLH | H | H | 5.06 | 0.23 |
| 2DSL | A | 32 | 8 | LDLHGTAH | H | H | 5.07 | 0.23 |
| 2BME | A | 152 | 8 | TSALTGEN | E | H | 5.09 | 0.24 |
| 2QF7 | A | 778 | 8 | SGNTSQPC | H | H | 5.11 | 0.24 |
| 1KG7 | A | 191 | 8 | ICTRSKPK | H | H | 5.12 | 0.24 |
| 1CC1 | S | 250 | 8 | RWNNGINW | H | H | 5.13 | 0.24 |
| 2OV9 | A | 123 | 8 | QGPPGHVH | H | H | 5.13 | 0.24 |
| 1G12 | A | 103 | 8 | APTTGTDS | H | H | 5.17 | 0.24 |
| 2OAF | A | 20 | 8 | CDPAKIAY | H | H | 5.21 | 0.24 |
| 2NT3 | A | 54 | 8 | VDLSAGQN | E | H | 5.22 | 0.24 |
| 1WVU | A | 231 | 8 | QNGPGTMT | H | H | 5.23 | 0.24 |
| 1KHV | A | 137 | 8 | HVKDGVMD | H | H | 5.24 | 0.24 |
| 3ES3 | A | 41 | 8 | FDRDDVAL | H | H | 5.28 | 0.24 |
| 1RXD | A | 103 | 8 | CVAGLGRA | E | H | 5.33 | 0.25 |
| 1Z6O | A | 62 | 8 | FNNYQTNR | H | H | 5.35 | 0.25 |
| 2FUJ | A | 19 | 8 | MDSMGHVN | H | H | 5.38 | 0.25 |
| 1Z2I | A | 249 | 8 | AVLTGMRL | H | H | 5.39 | 0.25 |
| 2HLJ | A | 16 | 8 | VDYNGHLR | H | H | 5.47 | 0.25 |
| 1MR1 | C | 237 | 8 | YSSPSAAC | H | E | 5.50 | 0.25 |
| 1AFV | B | 118 | 8 | MTHNPPIP | H | H | 5.53 | 0.26 |
| 1Z68 | A | 700 | 8 | TADDNVHF | E | H | 5.62 | 0.26 |
| 2QG7 | A | 162 | 8 | YNKNIAKK | H | E | 5.63 | 0.26 |
| 1P59 | A | 188 | 8 | HCKAQSPQ | H | H | 5.68 | 0.26 |
| 2A5Y | B | 437 | 8 | LSKRGALL | H | E | 5.70 | 0.26 |
| 1MVL | A | 81 | 8 | WNKIGDPV | H | H | 5.71 | 0.26 |
| 2F1N | A | 137 | 8 | AIAMRNND | E | H | 5.74 | 0.27 |
| 2FN3 | A | 398 | 8 | CAAGGLGF | E | H | 5.76 | 0.27 |
| 2DCM | A | 676 | 8 | AIDPVVVW | E | H | 5.77 | 0.27 |
| 2FNO | A | 12 | 8 | YWPVPFRG | E | H | 5.77 | 0.27 |
| 2RDM | A | 56 | 8 | IRFCQPPD | E | H | 5.81 | 0.27 |
| 1LNS | A | 51 | 8 | LQATAELD | H | H | 5.81 | 0.27 |
| 1EWK | A | 432 | 8 | CPGHVGLC | H | H | 5.82 | 0.27 |
| 1VC4 | A | 86 | 9 | TEPHRFGGS | E | H | 4.02 | 0.17 |
| 3BZ1 | C | 215 | 9 | KSPFGGEGW | H | H | 4.45 | 0.18 |
| 1ILE | A | 388 | 9 | HCWRCSTPL | E | E | 4.46 | 0.18 |
| 1JDP | A | 15 | 9 | PQDDSYLFS | E | H | 4.65 | 0.19 |
| 1XWS | A | 97 | 9 | SSGFSGVIR | H | E | 4.75 | 0.20 |
| 1VB3 | A | 98 | 9 | LFHGPTLAF | E | H | 4.76 | 0.20 |
| 1VI9 | A | 10 | 9 | SHVVYGHAG | E | H | 4.82 | 0.20 |
| 1SU8 | A | 153 | 9 | DFHEKDTPV | H | H | 5.03 | 0.21 |
| 2VA1 | A | 16 | 9 | KQNDSSIID | H | H | 5.07 | 0.21 |
| 2IUX | A | 275 | 9 | LGNMWAQTW | H | H | 5.10 | 0.21 |
| 2DE6 | A | 67 | 9 | DRCLHRGVQ | E | H | 5.18 | 0.21 |
| 3DI5 | A | 33 | 9 | QEIAPGHWT | H | H | 5.21 | 0.22 |
| 2IP2 | A | 87 | 9 | LLRDVEGSF | H | H | 5.31 | 0.22 |
| 1N1B | A | 176 | 9 | KNEKGIDFK | H | H | 5.31 | 0.22 |
| 1PIG | A | 124 | 9 | REFPAVPYS | H | H | 5.35 | 0.22 |
| 1I1J | A | 13 | 9 | ADQECSHPI | E | E | 5.46 | 0.23 |
| 1XF1 | A | 810 | 9 | HRHANGKPY | E | E | 5.47 | 0.23 |
| 1RZ2 | A | 140 | 9 | HRMKDGSMF | E | H | 5.49 | 0.23 |
| 1SQJ | A | 344 | 9 | SPSNLEGNW | H | E | 5.54 | 0.23 |
| 2V8V | A | 344 | 9 | IKINDGGAL | H | E | 5.55 | 0.23 |
| 1BJF | A | 53 | 9 | GNFFPYGDA | H | H | 5.59 | 0.23 |
| 2OK5 | A | 418 | 9 | ASKKDNEQH | H | E | 5.62 | 0.23 |
| 1R5T | A | 25 | 9 | SYSPYSHFR | H | E | 5.63 | 0.23 |
| 2ORW | A | 112 | 9 | DLTHKQNPF | E | H | 5.73 | 0.24 |
| 3C2G | A | 664 | 9 | VIDENRKEN | H | H | 5.76 | 0.24 |
| 1BRW | A | 111 | 9 | GRGLGHTGG | E | H | 5.77 | 0.24 |
| 1T3I | A | 150 | 9 | QLDEQESFD | E | H | 5.81 | 0.24 |
| 2ZYL | A | 65 | 9 | GYCRHMGGD | E | H | 5.81 | 0.24 |
| 1Z8X | A | 9 | 9 | EPFGGEKIN | E | H | 5.82 | 0.24 |
| 1O7D | A | 87 | 10 | GIYNNIQPAG | H | H | 3.51 | 0.13 |
| 2E8Y | A | 473 | 10 | GNTFHLKATG | H | H | 3.59 | 0.13 |
| 1FF9 | A | 384 | 10 | GAPIGSGGYS | E | H | 3.82 | 0.14 |
| 1H9A | A | 290 | 10 | GAGDSADFKP | E | H | 4.02 | 0.15 |
| 2QQM | A | 502 | 10 | GGKHRENKVF | E | E | 4.04 | 0.15 |
| 2Q40 | A | 249 | 10 | NELPSINDIT | E | H | 4.26 | 0.15 |
| 1I6I | A | 108 | 10 | MGKQEKDQQG | H | H | 4.36 | 0.16 |
| 1I0A | A | 435 | 10 | EQYTAVGGTA | H | H | 4.38 | 0.16 |
| 2Z3I | A | 45 | 10 | NVYHFTGGPC | E | H | 4.45 | 0.16 |
| 1G66 | A | 11 | 10 | RETTASPGYG | E | H | 4.50 | 0.16 |
| 3F2V | A | 116 | 10 | YRADGAVGCS | H | H | 4.53 | 0.16 |
| 1XRS | B | 142 | 10 | NMKGYAGHYG | H | H | 4.66 | 0.17 |
| 3G0T | A | 125 | 10 | NRTHKNREYG | H | E | 4.67 | 0.17 |
| 2FFJ | A | 187 | 10 | RGRPIISDAT | E | H | 4.72 | 0.17 |
| 2D1S | A | 443 | 10 | LIKYKGYQVP | H | H | 4.88 | 0.18 |
| 1OMW | A | 247 | 10 | STGDCPFIVC | H | E | 4.88 | 0.18 |
| 1TZ7 | A | 360 | 10 | AFYDKESEHL | H | H | 4.88 | 0.18 |
| 1DP4 | A | 10 | 10 | PLTNTSYPWS | E | H | 4.90 | 0.18 |
| 1UWC | A | 176 | 10 | FQVSSPETTQ | H | E | 4.95 | 0.18 |
| 2DE0 | X | 245 | 10 | SQNWRYATGG | E | H | 4.96 | 0.18 |
| 1RJ9 | A | 137 | 10 | AGDTFRAAGG | E | H | 4.98 | 0.18 |
| 1I4N | A | 82 | 10 | TEKHYFKGDP | E | H | 5.02 | 0.18 |
| 1K7C | A | 188 | 10 | YFPIDHTHTS | H | H | 5.04 | 0.18 |
| 1Y0P | A | 71 | 10 | CHSAHEKSMV | H | H | 5.05 | 0.18 |
