# Supplementary material for: How long is a piece of loop?
Source: PeerJ. 2013 Feb 12;1:e1. doi: 10.7717/peerj.1 (PMC3628373; doi:10.7717/peerj.1)
Supplement: Table S3 — This test set only consists of highly stretched loops (λ > 0.95). N and C columns are secondary structure elements (E: strand, H: helix). [file peerj-01-1-s005.docx]

| Code | Chain | Start | Length | Sequence | N | C | Span | Stretch |
| --- | --- | --- | --- | --- | --- | --- | --- | --- |
| 2ETX | A | 1960 | 6 | GFFLPP | H | H | 14.78 | 0.95 |
| 1NQ7 | A | 238 | 6 | AWQTHT | H | H | 14.78 | 0.95 |
| 1M0S | A | 174 | 6 | NFSILN | E | H | 14.79 | 0.95 |
| 1DJ0 | A | 197 | 6 | NAFVHH | E | H | 14.79 | 0.95 |
| 1YHV | A | 438 | 6 | RKAYGP | H | H | 14.79 | 0.95 |
| 1T15 | A | 1845 | 6 | YQCQEL | H | H | 14.79 | 0.95 |
| 1EG7 | A | 1097 | 6 | REPSLG | E | H | 14.80 | 0.95 |
| 1C0F | A | 217 | 6 | AYVALD | H | H | 14.81 | 0.95 |
| 2B94 | A | 161 | 6 | KIIPTR | H | H | 14.83 | 0.95 |
| 3DA4 | A | 151 | 6 | IGLKIS | H | H | 14.87 | 0.96 |
| 2I9E | A | 30 | 6 | GPLNQD | H | E | 14.92 | 0.96 |
| 4CMS | A | 52 | 6 | NHQRFD | H | H | 15.00 | 0.96 |
| 1SVT | A | 135 | 6 | SVPCSD | H | H | 15.02 | 0.97 |
| 3EG7 | A | 137 | 6 | CGFVEE | H | E | 15.04 | 0.97 |
| 1W88 | A | 334 | 6 | ETPKQK | H | H | 15.05 | 0.97 |
| 2QIO | A | 191 | 6 | GPIRTL | E | H | 15.09 | 0.97 |
| 2VHX | A | 360 | 6 | GVPFTE | H | H | 15.18 | 0.98 |
| 1F20 | A | 1167 | 6 | LSLLQP | H | E | 15.28 | 0.98 |
| 1VLB | A | 307 | 6 | LPAYMS | E | H | 15.29 | 0.98 |
| 2VBF | A | 474 | 6 | DIPMWN | H | H | 15.30 | 0.98 |
| 1XDY | A | 216 | 6 | RERPPT | E | H | 15.30 | 0.98 |
| 2IP6 | A | 72 | 6 | DKLHFT | H | H | 15.34 | 0.99 |
| 2EB1 | A | 16 | 6 | NYRFKN | H | H | 15.41 | 0.99 |
| 1DJE | A | 264 | 6 | YSTSMP | H | H | 15.44 | 0.99 |
| 2FQ6 | A | 240 | 6 | MGQMVD | H | H | 15.46 | 0.99 |
| 1N0U | A | 558 | 6 | PPVVAY | E | E | 15.47 | 0.99 |
| 1XYN | A | 84 | 6 | NYPAQG | E | E | 15.49 | 1.00 |
| 1S5J | A | 467 | 6 | NWLIPL | H | H | 15.50 | 1.00 |
| 1Z28 | A | 211 | 6 | VGRSLP | H | H | 15.50 | 1.00 |
| 1GK2 | A | 308 | 7 | NAVSDNP | H | E | 17.24 | 0.95 |
| 3DRW | A | 386 | 7 | KVPFNER | H | H | 17.25 | 0.95 |
| 3EJ5 | X | 195 | 7 | NRRSAPD | H | H | 17.29 | 0.95 |
| 2EAD | A | 133 | 7 | GNYLNWG | H | E | 17.33 | 0.96 |
| 1RK6 | A | 249 | 7 | SHHKVMG | E | H | 17.34 | 0.96 |
| 2OOL | A | 135 | 7 | PRDESRY | E | H | 17.38 | 0.96 |
| 2I9D | A | 101 | 7 | RFPYHND | E | H | 17.40 | 0.96 |
| 1MRG | A | 236 | 7 | NIQLLLN | H | H | 17.42 | 0.96 |
| 3DRA | B | 45 | 7 | IQKFQFT | H | H | 17.42 | 0.96 |
| 1G1Y | A | 121 | 7 | VFTTPEW | H | H | 17.54 | 0.97 |
| 1QP9 | A | 93 | 7 | CHYMEQT | H | H | 17.54 | 0.97 |
| 1UC8 | A | 252 | 7 | HTMEFKN | E | H | 17.57 | 0.97 |
| 1XV2 | A | 219 | 7 | AKIDYKD | H | H | 17.61 | 0.97 |
| 3DGK | A | 231 | 7 | AVNYEFE | H | H | 17.61 | 0.97 |
| 1NM1 | A | 196 | 7 | RGYSFTT | H | H | 17.66 | 0.97 |
| 2A5D | B | 34 | 7 | GTQMNIN | H | H | 17.80 | 0.98 |
| 3EEZ | A | 316 | 7 | CHAVWAC | H | H | 17.80 | 0.98 |
| 2CUY | A | 77 | 7 | AGGKPPA | H | E | 17.81 | 0.98 |
| 3DDS | A | 482 | 7 | KTNGITP | E | H | 17.82 | 0.98 |
| 1EEX | A | 17 | 7 | DGFVKEW | H | H | 17.84 | 0.98 |
| 1YQ2 | A | 764 | 7 | LWRAPTD | E | H | 17.84 | 0.98 |
| 3CNJ | A | 381 | 7 | KNPQRGT | E | E | 17.89 | 0.99 |
| 1T1U | A | 113 | 7 | ARQHFQD | E | H | 17.91 | 0.99 |
| 2YY7 | A | 293 | 7 | DWKHTFD | H | H | 17.94 | 0.99 |
| 1BGX | T | 395 | 7 | GGEWTEE | H | H | 17.95 | 0.99 |
| 3C9A | A | 315 | 7 | HCRCPRN | E | E | 17.96 | 0.99 |
| 3CMM | A | 860 | 7 | GRIIPAI | H | H | 18.00 | 0.99 |
| 2FSQ | A | 175 | 7 | YEFHITL | H | E | 18.04 | 0.99 |
| 2JG1 | A | 104 | 7 | EQGPEID | E | H | 18.07 | 1.00 |
| 1H8P | A | 66 | 7 | YAKCVFP | H | E | 18.36 | 1.01 |
| 2RFP | A | 79 | 7 | KETYPVT | H | H | 18.43 | 1.02 |
| 2G84 | A | 19 | 7 | EPRVLAA | H | H | 18.59 | 1.03 |
| 3D6R | A | 100 | 7 | REWYMLM | H | E | 18.68 | 1.03 |
| 3CNJ | A | 155 | 7 | LRVNHID | H | H | 18.70 | 1.03 |
| 1AEC | A | 43 | 7 | GVLISLS | H | H | 18.79 | 1.04 |
| 3E0K | A | 414 | 7 | QGFYEVG | H | H | 18.84 | 1.04 |
| 3DBH | B | 207 | 8 | YPPQVNFP | H | H | 20.53 | 0.95 |
| 1QLM | A | 127 | 8 | DYEDDADV | H | E | 20.55 | 0.95 |
| 1VJG | A | 155 | 8 | DVPYLDVF | H | H | 20.55 | 0.95 |
| 3ELJ | A | 263 | 8 | RPKYAGYS | H | H | 20.57 | 0.95 |
| 2AN0 | A | 315 | 8 | AGRKLTGD | H | H | 20.61 | 0.95 |
| 3GED | A | 82 | 8 | NACRGSKG | E | H | 20.63 | 0.96 |
| 1S48 | A | 201 | 8 | KHTYGEVT | H | H | 20.66 | 0.96 |
| 1QTN | A | 255 | 8 | HSIRDRNG | H | H | 20.66 | 0.96 |
| 1SQI | A | 285 | 8 | GMEFLAVP | H | H | 20.67 | 0.96 |
| 3CF6 | E | 970 | 8 | RQLNVIDN | H | H | 20.67 | 0.96 |
| 1PJT | A | 209 | 8 | SEPLDHRG | H | E | 20.69 | 0.96 |
| 1CIP | A | 176 | 8 | RTRVKTTG | H | E | 20.70 | 0.96 |
| 3H5Q | A | 419 | 8 | DHVVSPTL | E | E | 20.77 | 0.96 |
| 1RLZ | A | 252 | 8 | NPGLVLDI | H | H | 20.82 | 0.96 |
| 3DM7 | A | 142 | 8 | EPVPIEWP | E | H | 20.83 | 0.96 |
| 1X2G | A | 245 | 8 | FGQAPAFS | H | E | 20.84 | 0.96 |
| 2GP4 | A | 565 | 8 | RTATEIDL | H | H | 20.86 | 0.97 |
| 2BII | A | 91 | 8 | HGPVPYVP | E | H | 20.87 | 0.97 |
| 1W6Q | A | 1075 | 8 | AVFPFQPG | E | E | 20.98 | 0.97 |
| 1NO7 | A | 899 | 8 | HNMAERTT | H | E | 21.00 | 0.97 |
| 2RCN | A | 280 | 8 | EFGLWHLE | H | H | 21.02 | 0.97 |
| 1I4N | A | 23 | 8 | LIVQRRNH | H | H | 21.05 | 0.97 |
| 3CUX | A | 403 | 8 | EVPVGTIT | H | H | 21.14 | 0.98 |
| 1VYS | X | 339 | 8 | KAELNPQR | H | H | 21.18 | 0.98 |
| 1T5O | A | 279 | 8 | VVIEERPR | H | H | 21.19 | 0.98 |
| 2R14 | A | 344 | 8 | GAALNEPD | H | H | 21.20 | 0.98 |
| 2HSJ | A | 128 | 8 | SILPVNER | E | H | 21.23 | 0.98 |
| 3CJE | A | 148 | 8 | GQANTIRH | H | E | 21.29 | 0.99 |
| 1MTY | D | 514 | 8 | RLNCVFKN | H | H | 21.30 | 0.99 |
| 2FK6 | A | 152 | 8 | KDVPGSLK | E | H | 21.34 | 0.99 |
| 3EUH | A | 18 | 8 | NDFSISLP | H | H | 21.39 | 0.99 |
| 1FC9 | A | 247 | 8 | RQKVTINP | E | E | 21.40 | 0.99 |
| 2GOU | A | 338 | 8 | GYPLAEHV | H | H | 21.41 | 0.99 |
| 2IP4 | A | 313 | 8 | TRLSWKEG | H | E | 21.42 | 0.99 |
| 1JJ2 | O | 52 | 8 | KDKKGNSR | E | H | 21.42 | 0.99 |
| 2VWJ | A | 284 | 8 | GQPKEKVY | H | H | 21.43 | 0.99 |
| 1TQ8 | A | 125 | 8 | VGNVGLST | E | H | 21.43 | 0.99 |
| 3HB6 | A | 218 | 8 | QQGIKNLM | E | H | 21.44 | 0.99 |
| 1JYO | E | 114 | 8 | RLAVQITE | H | H | 21.49 | 0.99 |
| 1F89 | A | 147 | 8 | TLSPGEKS | H | E | 21.49 | 1.00 |
| 2BDW | A | 228 | 8 | GAYDYPSP | H | H | 21.51 | 1.00 |
| 1QO7 | A | 38 | 8 | SKIAPPTY | H | H | 21.52 | 1.00 |
| 2VDR | B | 251 | 8 | DAKTHIAL | E | H | 21.54 | 1.00 |
| 2GZA | A | 121 | 8 | KPSFTRRT | E | H | 21.59 | 1.00 |
| 2JE8 | A | 556 | 8 | FGFQSFPE | E | H | 21.67 | 1.00 |
| 1Z8X | A | 181 | 8 | GQVPPSMS | H | H | 21.70 | 1.00 |
| 3BU2 | A | 17 | 8 | QINPTEGE | E | E | 21.83 | 1.01 |
| 2BKA | A | 52 | 8 | RRKLTFDE | E | H | 21.88 | 1.01 |
| 1NG2 | A | 199 | 8 | KAKRGWIP | E | H | 21.93 | 1.02 |
| 2IUF | A | 232 | 8 | LQGKASFV | E | H | 22.06 | 1.02 |
| 1ZHX | A | 354 | 8 | GISWQRRW | H | E | 22.29 | 1.03 |
| 2GUP | A | 92 | 8 | QLPVHLEN | H | H | 22.36 | 1.04 |
| 2BPA | 1 | 352 | 8 | RYAPSYVS | H | H | 22.39 | 1.04 |
| 1GK9 | A | 123 | 8 | GFTPKRWE | H | H | 22.67 | 1.05 |
| 1QGC | 1 | 19 | 8 | GGETQVQR | H | H | 23.10 | 1.07 |
| 1LTK | A | 275 | 9 | KNVQIFLPV | H | E | 23.06 | 0.95 |
| 1TLV | A | 110 | 9 | KGLDIKNAL | H | H | 23.10 | 0.96 |
| 2PKJ | A | 317 | 9 | EPVADPYDQ | H | H | 23.18 | 0.96 |
| 2E18 | A | 242 | 9 | RRLPIGPSF | H | H | 23.19 | 0.96 |
| 2W5Y | A | 3800 | 9 | EVQLKSARR | H | H | 23.31 | 0.96 |
| 1YVF | A | 76 | 9 | STVKAKLLS | H | H | 23.37 | 0.97 |
| 2AG5 | A | 81 | 9 | VAGFVHHGT | E | H | 23.46 | 0.97 |
| 2V26 | A | 276 | 9 | RGCTRYFAN | H | H | 23.46 | 0.97 |
| 3DNU | A | 328 | 9 | TPFYDIISA | E | H | 23.51 | 0.97 |
| 1OFL | A | 372 | 9 | RLKFETPHQ | H | E | 23.51 | 0.97 |
| 1TY7 | B | 181 | 9 | KTTCLPMFG | H | E | 23.69 | 0.98 |
| 1V7M | X | 109 | 9 | GTQLPPQGR | H | E | 23.71 | 0.98 |
| 1ZMT | A | 80 | 9 | DIFAPEFQP | E | H | 23.73 | 0.98 |
| 2ERK | A | 264 | 9 | SLPHKNKVP | H | H | 23.75 | 0.98 |
| 1MJO | A | 21 | 9 | VKKITVSIP | H | H | 23.91 | 0.99 |
| 1R89 | A | 255 | 9 | KPKHPLEIE | H | H | 23.94 | 0.99 |
| 2F7F | A | 196 | 9 | GIPVSGTHA | H | H | 23.96 | 0.99 |
| 3E03 | A | 98 | 9 | NASAIWLRG | E | H | 23.98 | 0.99 |
| 2I1O | A | 173 | 9 | DQDPVGTMP | H | H | 24.05 | 0.99 |
| 1RQP | A | 187 | 9 | IVRFNRPAV | H | E | 24.13 | 1.00 |
| 2UUU | A | 86 | 9 | IHVDPPKQY | H | H | 24.30 | 1.00 |
| 3GBE | A | 385 | 9 | GMTNYPFKA | H | H | 24.31 | 1.01 |
| 2EW8 | A | 89 | 9 | NAGIYPLIP | E | H | 24.34 | 1.01 |
| 1QQP | 1 | 19 | 9 | GGETQIQRR | H | H | 24.64 | 1.02 |
| 1OSY | A | 14 | 9 | KKIDFDYTP | H | E | 24.71 | 1.02 |
| 2V6G | A | 349 | 9 | GNECFLDSM | H | H | 24.78 | 1.02 |
| 3D7L | A | 62 | 9 | ATGSATFSP | E | H | 24.86 | 1.03 |
| 1DPC | A | 407 | 9 | GEIEEVPMT | H | H | 25.31 | 1.05 |
| 1YBV | A | 114 | 9 | NSGVVSFGH | E | H | 25.87 | 1.07 |
| 1UKC | A | 238 | 10 | SSFWPTQRTV | E | H | 26.29 | 0.95 |
| 1SH0 | A | 96 | 10 | QTIDPPDKWS | H | H | 26.34 | 0.95 |
| 1UYR | A | 1623 | 10 | ANSGARIGMA | E | H | 26.35 | 0.95 |
| 2Z8F | A | 383 | 10 | NMGDFTYMPG | H | H | 26.41 | 0.96 |
| 1YB1 | A | 115 | 10 | NAGVVYTSDL | E | H | 26.45 | 0.96 |
| 1O2D | A | 311 | 10 | LGLYEKVAVS | H | H | 26.54 | 0.96 |
| 3CKJ | A | 313 | 10 | WPVSLADRPP | E | H | 26.57 | 0.96 |
| 1GZ6 | A | 98 | 10 | NNAGILRDRS | E | H | 26.60 | 0.96 |
| 1SQH | A | 164 | 10 | CAKVPDLPSE | H | E | 26.75 | 0.97 |
| 1KKE | A | 290 | 10 | RSTSPNLRYP | E | E | 26.80 | 0.97 |
| 2BLN | A | 182 | 10 | HGNILEIAQR | H | H | 26.88 | 0.97 |
| 1NY5 | A | 134 | 10 | LKEEEYVFES | H | H | 26.94 | 0.97 |
| 2FBY | A | 42 | 10 | DDLPFLEFTG | H | E | 26.95 | 0.97 |
| 2BIB | A | 304 | 10 | YKPIPSFQAG | H | E | 27.07 | 0.98 |
| 2DY1 | A | 373 | 10 | VPFARLPDPN | H | E | 27.12 | 0.98 |
| 2DJI | A | 184 | 10 | LRKYAPIAPA | H | H | 27.15 | 0.98 |
| 1IDJ | A | 317 | 10 | CSGTFSEDST | E | H | 27.24 | 0.99 |
| 1XV2 | A | 141 | 10 | RRQPEEKRQD | H | E | 27.34 | 0.99 |
| 2DJF | B | 344 | 10 | GPMAVAFEVY | H | H | 27.48 | 0.99 |
| 1JA9 | A | 113 | 10 | SNSGMEVWCD | E | H | 27.85 | 1.01 |
| 1YG8 | A | 122 | 10 | HQPLGGYQGQ | E | H | 27.93 | 1.01 |
| 2FGT | A | 230 | 10 | KKEFVTESIK | E | H | 27.99 | 1.01 |
| 1UP8 | A | 28 | 10 | RGVVPSFANG | H | H | 28.05 | 1.01 |
| 2YVL | A | 214 | 10 | EILHRHYKTI | E | H | 28.67 | 1.04 |
